# Integrated Analysis of scRNA-Seq and Bulk RNA-Seq Reveals Metabolic Reprogramming of Liver Cancer and Establishes a Prognostic Risk Model

**DOI:** 10.3390/genes15060755

**Published:** 2024-06-08

**Authors:** Zhuang Xiong, Lizhi Li, Guoliang Wang, Lei Guo, Shangyi Luo, Xiangwen Liao, Jingfeng Liu, Wenhao Teng

**Affiliations:** 1 Department of Hepatopancreatobiliary Surgery, Clinical Oncology School of Fujian Medical University, Fujian Cancer Hospital, Fuzhou 350014, China; xiongzhuang@fzu.edu.cn; 2 Interdisciplinary Institute for Medical Engineering, Fuzhou University, Fuzhou 350108, China; ganklei@fzu.edu.cn (L.G.); luoshangyi@fzu.edu.cn (S.L.); liaoxw@fzu.edu.cn (X.L.); 3 Department of Pediatric Surgery, Shengli Clinical Medical College of Fujian Medical University, Fuzhou 350001, China; lilizhi@fjmu.edu.cn; 4 CAS Key Laboratory of Genome Sciences and Information, Beijing Institute of Genomics, Chinese Academy of Sciences/China National Center for Bioinformation, Beijing 100101, China; wangguoliang@big.ac.cn

**Keywords:** liver cancer, metabolic reprogramming, single-cell RNA-seq, prognostic risk model

## Abstract

Liver cancer manifests as a profoundly heterogeneous malignancy, posing significant challenges in terms of both therapeutic intervention and prognostic evaluation. Given that the liver is the largest metabolic organ, a prognostic risk model grounded in single-cell transcriptome analysis and a metabolic perspective can facilitate precise prevention and treatment strategies for liver cancer. Hence, we identified 11 cell types in a scRNA-seq profile comprising 105,829 cells and found that the metabolic activity of malignant cells increased significantly. Subsequently, a prognostic risk model incorporating tumor heterogeneity, cell interactions, tumor cell metabolism, and differentially expressed genes was established based on eight genes; this model can accurately distinguish the survival outcomes of liver cancer patients and predict the response to immunotherapy. Analyzing the immune status and drug sensitivity of the high- and low-risk groups identified by the model revealed that the high-risk group had more active immune cell status and greater expression of immune checkpoints, indicating potential risks associated with liver cancer-targeted drugs. In summary, this study provides direct evidence for the stratification and precise treatment of liver cancer patients, and is an important step in establishing reliable predictors of treatment efficacy in liver cancer patients.

## 1. Introduction

Malignant liver tumors, one of the most common solid tumors, are the second leading cause of cancer-related deaths worldwide, with their global incidence increasing annually [1,2]. Primary liver cancer (PLC) includes three main histological subtypes: hepatocellular carcinoma (HCC), intrahepatic cholangiocarcinoma (ICC), and hepatocellular and intrahepatic cholangiocarcinoma (CHC) [3,4], with HCC constituting 75% to 85%, and ICC representing 10% to 15%. Despite significant advances in the treatment of liver cancer over the past few decades [5], the 5-year survival rate of patients remains dishearteningly low, coupled with a high recurrence rate [6]. This poor prognosis may be attributed to the still limited understanding of the immune microenvironment, molecular heterogeneity, and metabolic events of liver tumors, as well as the lack of effective prognostic predictors and therapeutic targets [7,8,9].

The intricate connection between metabolic dysregulation and tumorigenesis has garnered increasing attention [10,11,12,13,14]. Alterations in metabolic pathways in tumors have become a driving force for cancer cells to acquire beneficial energy or evade detection by immune system [15]. Metabolic reprogramming events such as glycolysis and oxidative phosphorylation have been identified as hallmarks of cancer [16,17]. Mossmann et al. reported that *RBM39*-mediated upregulation of asparagine synthesis leads to enhanced arginine uptake, forming a positive feedback loop to maintain high arginine levels and carcinogenic metabolism [18]. Wu et al. found that changes in fatty acid metabolism caused by mitochondrial fission can promote liver cancer cell proliferation, metastasis, and tumor growth in vivo, suggesting that mitochondrial fission and its mediated reprogramming of fatty acid metabolism may become potential targets for liver cancer treatment [19]. Wu et al. used mass spectrometry to reveal that fatty acid oxidation (FAO) metabolism is active in liver cancer cells, which in turn promotes tumorigenesis by activating the transcription factor FOXM1 [20]. Cubuk et al. found the activity of many metabolic modules was significantly associated with prognosis at a greater magnitude than any of their constituent genes [21].

The application of multi-omics technology has revealed significant molecular heterogeneity in various human cancer types, portraying a complex ecosystem of malignant cells surrounded by non-malignant stroma, including myeloid cells, fibroblasts, endothelial cells, and T cells [22,23]. Malignant cells are likely pivotal contributors to this heterogeneity, as they possess the ability to interact with other cells, thereby fostering their growth. Stromal cells and immune cells within the tumor ecosystem not only potentially curb tumor cell growth and proliferation through immune system activation but also might influence tumor progression by engaging in competition with malignant cells for nutrients in the tumor ecosystem [24]. Consequently, malignant cells might acquire unique capabilities to reprogram the tumor microenvironment and evade this competition [25]. Prognostic risk models based on relevant biomarkers of tumor metabolic reprogramming events in malignant cells can thus more accurately predict immune responses and patient prognosis. In this study, we downloaded the scRNA-seq, RNA-seq, and corresponding clinical data of liver cancer patients from public databases. We constructed a single-cell atlas of liver cancer covering 11 cell types and found that the proportions of epithelial cells and fibroblasts were significantly increased in tumor tissues. By quantifying the metabolic activity of cell types, malignant cells were revealed to be significantly enriched in multiple metabolism-related pathways, indicating that metabolic reprogramming events occurred in malignant cells. Subsequently, a prognostic multigene signature featuring differentially expressed genes (DEGs) related to tumor cell metabolism was constructed in the TCGA cohort and validated in the International Cancer Genome Atlas (ICGC) cohort. Finally, we conducted additional analyses encompassing functional enrichment, immune infiltration, and drug sensitivity to determine the underlying mechanisms involved.

## 2. Materials and Methods

### 2.1. Single Cell Data Acquisition and Preprocessing

The scRNA-seq dataset GSE189903 for liver cancer was obtained from the GEO (https://www.ncbi.nlm.nih.gov/) (accessed on 25 December 2023) database, comprising 7 normal tissue samples and 27 tumor samples [25]. The raw data were processed using CellRanger (version 7.2.0), a tool employed for alignment, tagging, and quantification of genes and transcripts. Subsequently, the count matrix of each sample was analyzed with the Seurat package (version 4.9.9.9059) within the R software environment (version 4.3.0) [26,27]. Cells of low-quality were removed according to the following criteria: fewer than 300 UMIs, more than 20% mitochondrial reads, fewer than 300 expressed genes, or more than 7000 expressed genes. The cell cycle score was computed utilizing the CellCycleScoring function, and data standardization was executed via the ScaleData function, with specification of the “vars.to.regress” parameter to account for cell cycle heterogeneity. The DoubletFinder package (version 2.0) was used for the identification and removal of doublets in the sample.

### 2.2. Cell Clustering and Annotation

The FindVariableFeatures function was employed to identify the top 3000 highly variable genes (HVGs). Subsequently, the ScaleData function was applied to identify, center, and scale these HVGs. To eliminate batch effects, the Harmony package (1.2.0) was utilized to integrate the datasets [28]. For principal component analysis (PCA) dimensionality reduction on the aforementioned top 3000 highly variable genes, the RunPCA function was utilized. To improve clustering results, the resolution parameter was set to 0.5 in the configuration of the FindNeighbors and FindClusters functions. For identification of cluster marker genes, the Wilcoxon rank sum test was employed by the FindAllMarkers function, screening for *p* values less than 0.05 and log fold changes greater than 1. Based on the clustering results, different subclusters were first annotated using the singleR package (version 2.6.0) [29]. Use immune cell marker genes (*PTPRC*), stromal cell marker genes (*VWF* and *MGP*) and epithelial cell marker genes (*EPCAM* and *KRT19*) to first verify whether the three major cell types corresponding to the annotation results are correct. The annotation results were then further verified based on cell subtype marker genes reported in the liver cancer literature [24,30].

### 2.3. Copy Number Variation Analysis

The infercnv package (1.20.0) was employed for the identification of copy number variations (CNVs) in malignant cells. The subset function is used to extract gene expression data from reference cells (B cells and T cells) and malignant cells, utilizing the CreateInfercnvObject function to instantiate an object for CNV analysis. The infercnv::run function was used to identify the malignancy of malignant cells.

### 2.4. Gene Set Variation Analysis and Metabolic Activity Analysis

The GSVA package (1.52.2) and the msigdbr package (7.5.1) were employed for conducting the enrichment analysis of hallmark gene sets, aiming to investigate the most significantly enriched tumor-related biological states and processes of cell types [31]. Additionally, the scMetabolism package (0.2.1) was utilized for the quantification of metabolic activity for cell types [32].

### 2.5. Cell Communication Analysis

Cellular interaction networks were determined using the CellChat package (version 2.0) through the integration of the expression profiles of signaling ligands, receptors, and their respective cofactor genes [33]. The extraction of gene expression data and the derivation of cell type annotation results were accomplished through the utilization of the Subset function. Subsequently, a CellChat object was generated by employing the createCellChat function. The identification of overexpressed ligands or receptors within cells is achieved via the employ of the identifyOverExpressedGenes function. Concurrently, the identifyOverExpressedInteractions function exploits the overexpressed ligands or receptors to construct a protein-protein interaction (PPI) network. The modeling of the probability associated with cell-to-cell communication and the prognostication of cell communication networks were effectuated using the computeCommunProb function. Visualization of cell–cell interactions and related signaling pathways was facilitated by functions such as netVisual_circle, netVisual_aggregate, and netVisual_heatmap.

### 2.6. RNA-Seq Data Acquisition and Preprocessing

Bulk RNA-seq data for liver cancer, along with its clinicopathological characteristics, were obtained from the TCGA database [34]. The dataset, which included a count matrix comprising 418 samples and their corresponding clinical information, served as the test set for constructing a prognostic risk model. Additionally, to validate the risk model, RNA-seq data and clinical information for 231 liver cancer samples were retrieved from the ICGC database, forming an independent external cohort [35]. To mitigate batch effects, the Combat function of the SVA package was utilized, and normalization of the expression profile data was carried out through the application of the Limma package (3.60.2) [36,37].

### 2.7. Predictive Model Construction and Validation

First, potential prognostic DEGs were identified using univariate Cox regression analysis. Subsequently, the glmnet package (4.1-8) was employed to conduct LASSO regression analysis, screening genes exhibiting elevated correlations to avert overfitting [38]. Ultimately, the definitive prognostic risk model, grounded in tumor metabolic reprogramming events within malignant cells, was meticulously crafted through stepwise multivariate Cox regression analysis, yielding the formula for the risk score: risk score = ∑(coefficient of gene × expression of a gene). Receiver operating characteristic (ROC) curves were delineated to assess the predictive efficacy of genetic signatures for 1, 3, and 5 year overall survival (OS) in patients diagnosed liver cancer by the timeROC package (version 0.4) [39].

### 2.8. Immune Infiltration Analysis

CIBERSORTx, developed by the Alizadeh and the Newman Laboratory, was employed for the estimation of gene expression profiles. This approach facilitates the utilization of gene expression data to gauge the abundance of constituent cell types within heterogeneous cell populations [40]. Marker genes for cell types and expression profile data for liver cancer were extracted, and CIBERSORTx was used to ascertain the infiltration proportions of 22 distinct immune cell types in the context of liver cancer. Additionally, predictions of stromal cell and immune cell scores were conducted employing the ESTIMATE package (https://bioinformatics.mdanderson.org/estimate/rpackage.html). Furthermore, an examination of disparities in immune checkpoint-related gene expression between the high- and low-risk cohorts was performed utilizing the Wilcoxon test (*p* < 0.05).

### 2.9. Drug Sensitivity Analysis

To evaluate drug susceptibility in the high- and low-risk groups, drug susceptibility analysis was conducted using the ggpubr package (version 0.6,0), and IC50 calculations were performed using OncoPredict (version 1.2) [41]. The relationship between gene signatures and drug IC50 values was assessed through Pearson correlation analysis.

## 3. Results

**Figure 1:**
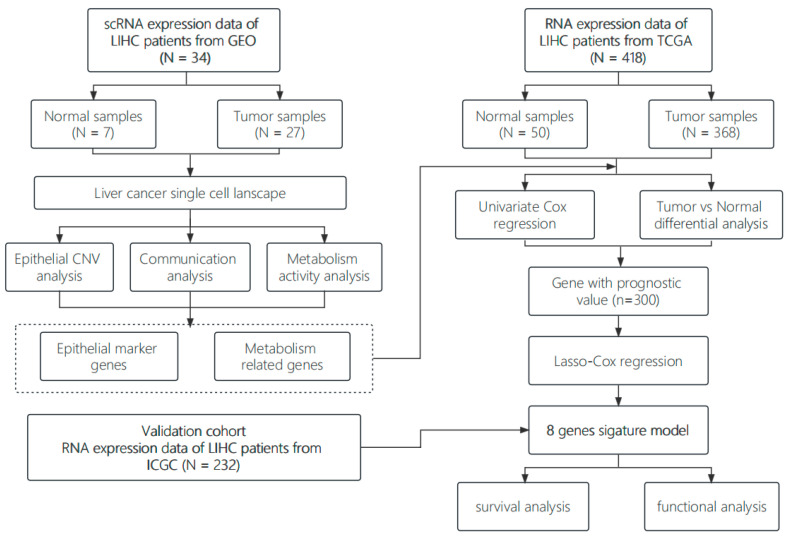
Schematic overview of the analysis workflow.

The flow chart of this study is shown in Figure 1. A total of 418 patients with liver cancer from the TCGA-LIHC cohort and 231 from the ICGC (LIRI-JP) cohort were included in the study.

### 3.1. Construction of a Single-Cell Atlas for Human Liver Cancer

**Figure 2:**
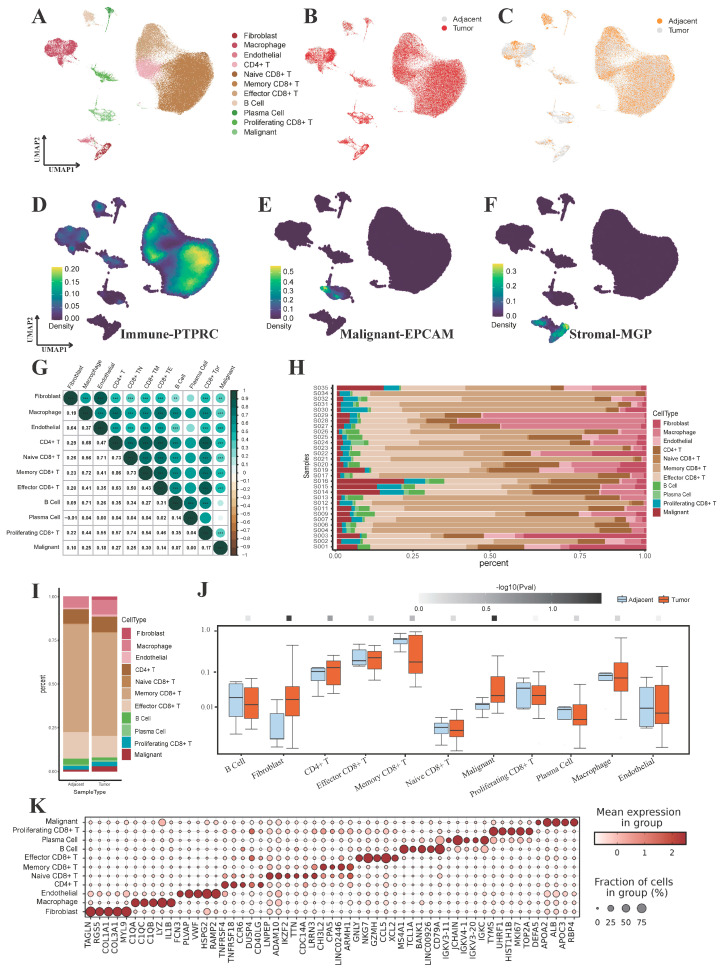
Construction of a single-cell atlas and determination of the cellular heterogeneity of liver cancer. (**A**) UMAP plot of the cell annotations. (**B**,**C**) UMAP plot of the cell distribution in tumor and adjacent tissues. (**D**–**F**) UMAP plots of marker genes of malignant cells, stromal cells and immune cells. (**G**) Heatmap of the correlations in expression between cell types (**: *p* < 0.01, ***: *p* < 0.001). (**H**) Stacked plot of the cell proportion distribution in different samples. (**I**) Stacked plot of the cell proportion distribution in tumor tissue and adjacent tissue. (**J**) Boxplots of cell proportions in tumor tissue and adjacent tissue. (**K**) Dot plot of the top 5 DEGs to cell type.

For an in-depth exploration of the cellular diversity and molecular characteristics of liver cancer tissues, we generated scRNA-seq profiles from 27 tumor tissue (tumor core area and edge area) and 7 adjacent non-tumor tissue integrated in public databases. Following rigorous quality control procedures, single-cell transcriptomes of a total of 105,829 cells from all samples were obtained for subsequent analysis. After post-normalization of gene expression for identifying highly variable genes, we applied PCA and Harmony to eliminate batches, followed by uniform manifold approximation and projection (UMAP) for dimensionality reduction and clustering. These cells were annotated into 11 major cell types based on classical markers, including fibroblasts (*ACTA2*), macrophages (*CD68*, *CD163* and *CSF1R*), endothelial cells (*PTPRB* and *PECAM1*), B cells (*MS4A1* and *CD79A*), plasma cells (*MZB1*, *IGKC* and *JCHAIN*), proliferating CD8+ T Cells (*CD27* and *CD44*), malignant cells (*EPCAM*, *KRT19* and *KRT7*), CD4+ T cells (*CD4*), naive CD8+ T cells (*CD8A*, *CD8B* and *LEF1*), memory CD8+ T cells (*CD8A*, *CD8B* and *IL7R*), and effector CD8+ T (*CD8A*, *CD8B*, *PRF1* and *KLRG1*) (Figure 2A–F,K). Calculation of the correlation of expression for each cell type revealed that there was a strong correlation between T cell subtypes and between stromal cell subtypes, thereby reinforcing the precision of cell identification (Figure 2G). Variations in the proportions of cell types among samples suggested substantial molecular heterogeneity in liver cancer samples (Figure 2H). Notably, there are also fewer malignant cells in the adjacent tissue, and the numbers of fibroblasts and malignant cells increased in the tumor tissue (Figure 2I). Significance analysis indicated that malignant cells exhibited the most significant increase in growth among the cell types (Figure 2J).

### 3.2. Inter-Transcriptome Heterogeneity of Human Liver Malignant Cells

**Figure 3:**
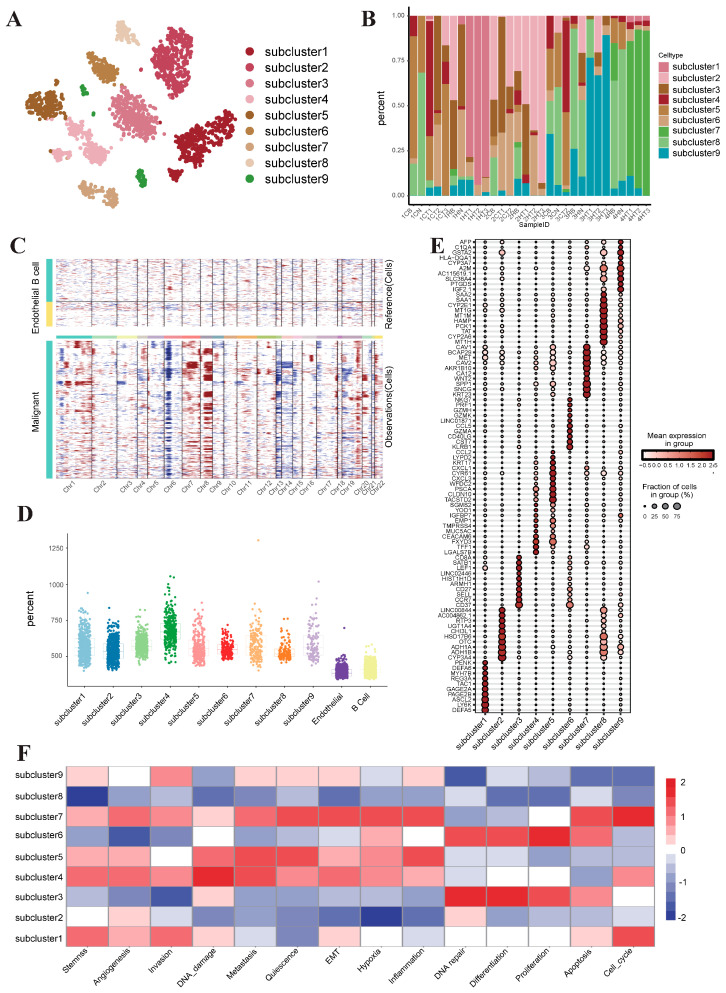
Transcriptomic heterogeneity across human liver malignant cells. (**A**) UMAP plot illustrating subclusters of malignant cells. (**B**) Stacked plot depicting the proportions of malignant cell subclusters across different samples. (**C**) Heatmap depicting CNV analysis of malignant cells. (**D**) Box plots showing CNV scores for malignant cell subclusters and reference cells. (**E**) Scatter plot highlighting the top 10 DEGs in malignant cell subclusters. (**F**) Heatmap of GSVA analysis of malignant cell subclusters.

All malignant cells were meticulously extracted and subsequently reclustered into nine principal subclusters (Figure 3A). Malignant cells exhibited a profound level of inter-tumor heterogeneity, aligning seamlessly with the findings of previous studies delineating the specificity of tumors in individual patients (Figure 3B). B cells and endothelial cells were employed as normal references for the analysis of copy number variations. The findings revealed that the mutations predominantly clustered on chromosome 7 and chromosome 8 (Figure 3C). The CNV scores of the nine subclusters exhibited a notable increase compared to those of the reference cells, thereby providing additional evidence confirming that all the extracted cells were indeed malignant cells (Figure 3D). Subcluster 1 highly expressed development-related genes (*ASCL2*, *PAGE2B*, *GAGE2A*, and *MYH7B*), which are enriched in cell repair, E2F targets and WNT signaling pathways. Subclusters 2 and 8 exhibited high expression of the metabolic factors *CYP3A4*, *OTC*, *PCK1* and *TAT*, suggesting that these genes may be involved in tumor cell metabolic reprogramming events. Enrichment analysis revealed that subclusters 2 and 8 were associated with oxidative phosphorylation, fatty acid metabolism and cholic acid metabolism. Subclusters 3 and 6 showed significant expression of immune regulatory genes (*CD37* and *CD27*) and cytotoxic genes (*GZMK* and *GZMH*), respectively. Subclusters 4 and 5 may be related to tumor invasion and metastasis (migration factors *CXCL3* and *CXCL1*; adhesion factors *CEACAM6* and *EMP1*), suggesting that they may be tumor cell clusters with a greater degree of malignancy. Similar to subcluster 1, subcluster 7 was involved in cell differentiation and tissue development (with high expression of *SPP1* and *WNT2*) (Figure 3E). Gene set variation analysis (GSVA) showed that multiple subclusters, such as tumor differentiation and proliferation (subcluster 3 and subcluster 6), cell cycle (subcluster 1 and subcluster 7), cell stemness and tumor invasion (subcluster 1, subcluster 4, subcluster 5, subcluster 7, and subcluster 9), share common activation characteristics (Figure 3F).

### 3.3. Metabolic Reprogramming of Malignant Cells in Human Liver Cancer

**Figure 4:**
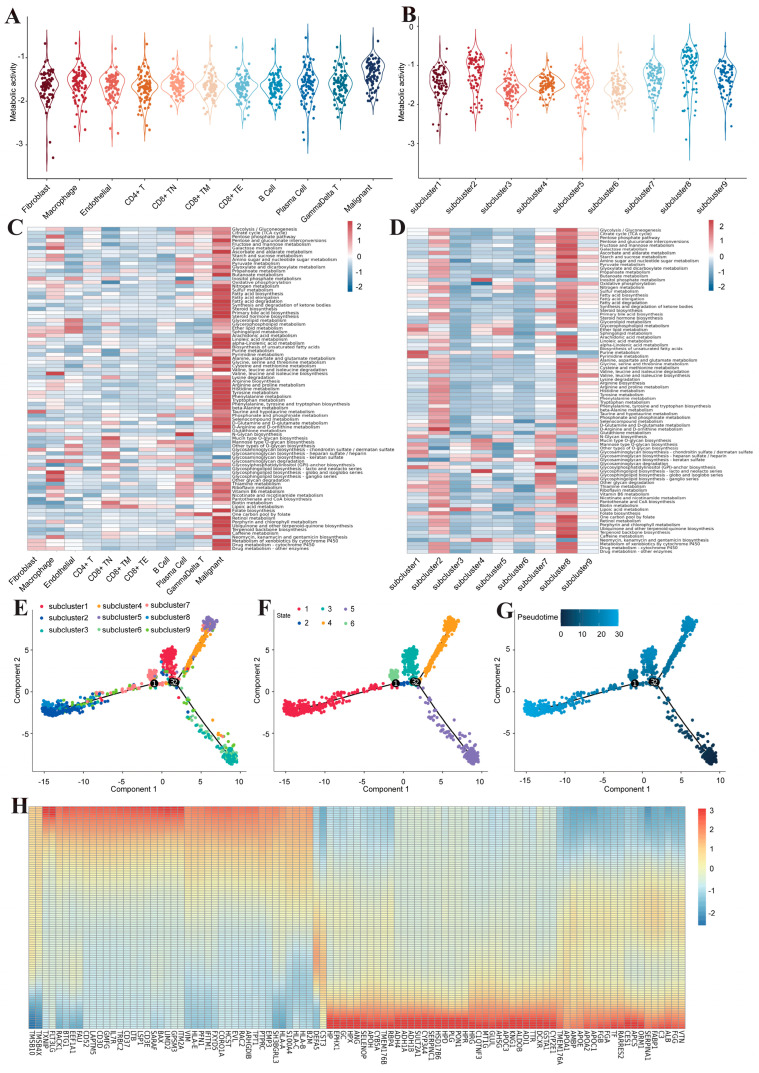
Metabolic reprogramming of malignant cells in human liver cancer. (**A**,**B**) Heat maps illustrating metabolic pathway enrichment for major cell types and malignant cell subclusters. (**C**,**D**) Quantitative violin plots demonstrating metabolic activity for major cell types and malignant cell subclusters. (**E**–**G**) Pseudotime trajectory differentiation plots for malignant cell subclusters (1, 2, 3 means branch point). (**H**) Heat map of differentially expressed genes along a pseudotime trajectory.

Considering that metabolic reprogramming is a hallmark of cancer, we performed a series of analyses on metabolic pathways in various cell types. We found that malignant cells exhibit significantly greater metabolic activity than other cell types (Figure 4A). High enrichment of oxidative phosphorylation, glycolysis, and the tricarboxylic acid cycle indicated significant mitochondrial activity in liver cancer metabolism (Figure 4C), suggesting that tumor cells are the driving force for reprogramming metabolic pathways to obtain energy or evade immune surveillance. We then quantified the metabolic activity of nine subclusters of malignant cells. Similar to the previous description, the enrichment of metabolic pathways in subclusters 2 and 8 was the most significant (Figure 4B,D). To further explore the dynamic transition process between subclusters of malignant cells, we used Monocle2 to conduct pseudotime trajectory analysis (Figure 4E–G). The analysis revealed that malignant cells existed in six different cell development states. Subclusters 3 and 9, which represent early stages of development, exhibited two differentiations. Through subcluster 1 as an intermediate transition state, one part differentiates into subcluster 4 and subcluster 5, and the other part differentiates into subcluster 2 and subcluster 8. From the perspective of pseudotime, subcluster 2 and subcluster 8 are located at the terminal stage of differentiation. Inspection of the genetic heatmap pertaining to cell fate transition elucidates that the highly expressed genes in the early phases of differentiation primarily included immune regulatory genes (*CD52*, *CD3D*, *CD37*, and *CD3E*), cell development genes (*GMFG* and *IL7R*), and antigen presentation genes (*HLA-E*, *HLA-B*, and *HLA-C*). Conversely, the highly expressed genes towards the culmination of differentiation predominantly included coagulation genes (*VTN*, *FGG*, *FGA*, and *FGB*) and metabolic genes (*FABP1*, *APOC1*, *APOA2*, *APOE*, *AMBP*, *APOA1*, *APOC3*, *ADH1B*, *ADH1A*, and *ADH4*) (Figure 4H). This further revealed that metabolic reprogramming occurs in malignant cells during the terminal differentiation stage in liver cancer, thereby sustaining tumor growth and facilitating metastasis.

### 3.4. The MIF/CD74 Axis Is Rich in Interactions between Malignant Cells and Other Cells

**Figure 5:**
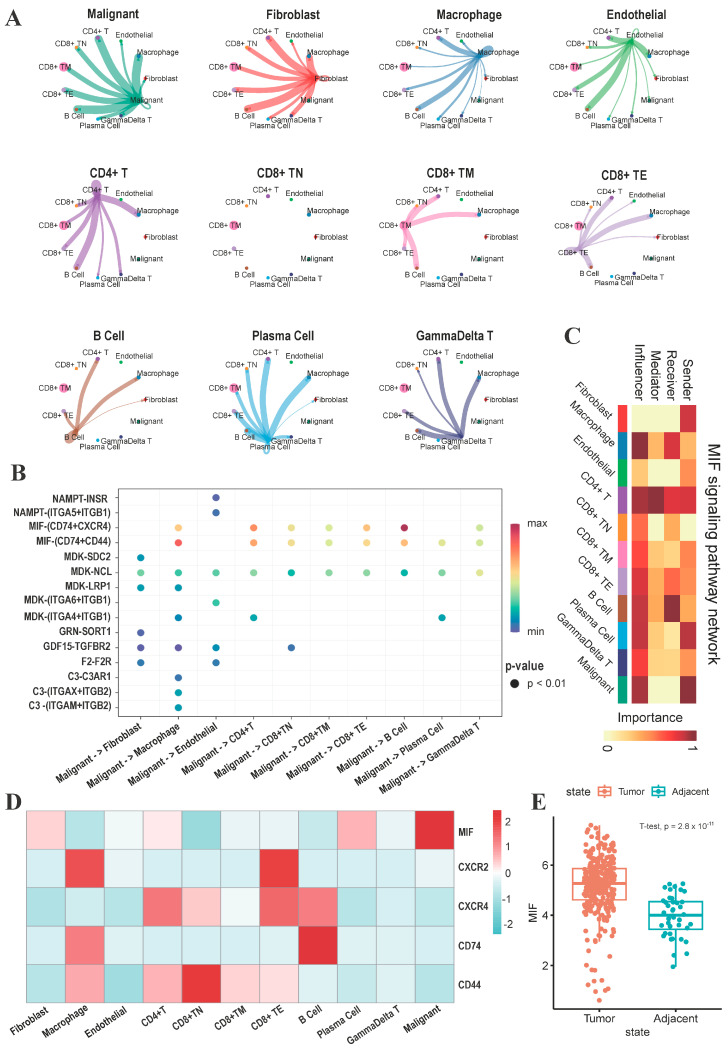
The MIF/CD74 axis is enriched in interactions between malignant cells and other cells. (**A**) Circular diagram representing signaling pathways originating from each cell type. (**B**) Dot plot highlighting significant interactions (L-R pairs) between malignant cells and other cell types. (**C**) Roles of cell types in the MIF signaling pathway. (**D**) Heatmap depicting the expression of key genes in the MIF signaling pathway across cell types. (**E**) Boxplot illustrating the expression of the MIF gene in tumor and adjacent tissues in TCGA-LIHC cohort.

To explore the intricate cellular interactions within the tumor microenvironment, we conducted cell–cell interaction analysis based on ligand–receptor pairs. Both malignant cells and fibroblasts exhibited pronounced interactions with other cell types (Figure 5A). We computed the signals emanating from malignant cells, which act as ligands for various cell types. Our analysis revealed an enrichment of interactions between malignant cells and other cells, particularly involving the MIF/CD74 axis (Figure 5B). The macrophage migration inhibitory factor (MIF), a cytokine pivotal in immune and inflammatory responses, assumed a central role in our investigation. The heatmap shows that malignant cells predominantly emit signals in the MIF signaling pathway, whereas B cells and tumor-associated macrophages primarily act as signaling recipients. Additionally, various other cell types function as intermediate mediators in this signaling network (Figure 5C). Examination of the key genes within the MIF signaling pathway revealed distinct expression patterns: MIF predominantly resides in malignant cells, CD74 manifests in B cells and TAMs, while CD44 is expressed across various subtypes of TAMs and T cells (Figure 5D). Analysis of the TCGA-LIHC cohort revealed significant upregulation MIF expression within tumor tissues (Figure 5E). These findings provide further evidence supporting the potential development of liver cancer immunotherapy through targeted intervention in the MIF/CD74 axis.

### 3.5. Identification and Functional Enrichment Analysis of DEGs in Liver Cancer RNA-Seq Data

**Figure 6:**
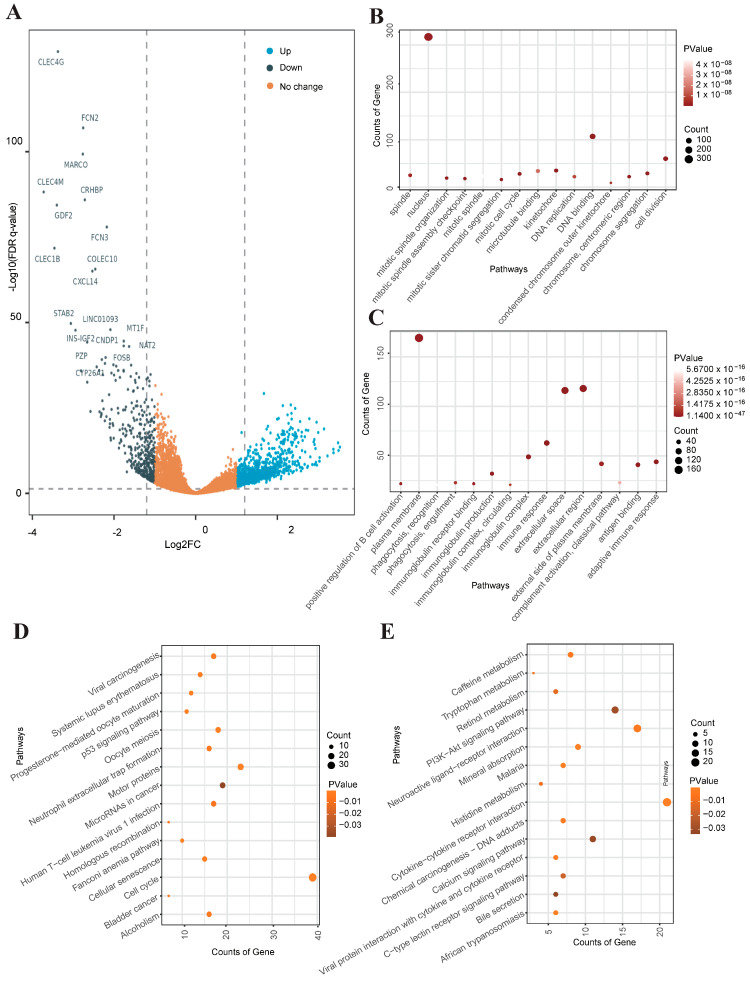
Identification and functional enrichment analysis of DEGs in liver cancer RNA-seq data. (**A**) Volcano plot of DEGs. (**B**,**C**) Gene ontology (GO) analysis of the differentially up- and down-regulated genes. (**D**,**E**) KEGG enrichment analysis of the differentially up- and down-regulated genes.

Through RNA-seq differential expression analysis of TCGA-LIHC cohort, a total of 1429 significantly differentially expressed genes (DEGs) were identified, including 1019 up-regulated genes and 410 down-regulated genes (Figure 6A). Gene Ontology (GO) enrichment analysis showed that cell cycle-related pathways (mitotic spindle organization, mitotic spindle, mitotic cell cycle) such as DNA replication and mitotic division were significantly up-regulated in tumor tissues and were closely associated with the rapid proliferation of malignant cells (Figure 6B,C). Additionally, KEGG enrichment analysis indicated that DEGs were primarily enriched in metabolic pathways, including histidine metabolism, tryptophan metabolism, and mineral metabolism, alongside the PI3K-Akt pathway. The PI3K-Akt pathway, an intracellular signal transduction cascade, orchestrates cellular responses to extracellular signals, fostering metabolism, proliferation, cell survival, growth, and angiogenesis (Figure 6D,E).

### 3.6. Construction and Validation of the Metabolism-Related Prognostic Risk Model

**Figure 7:**
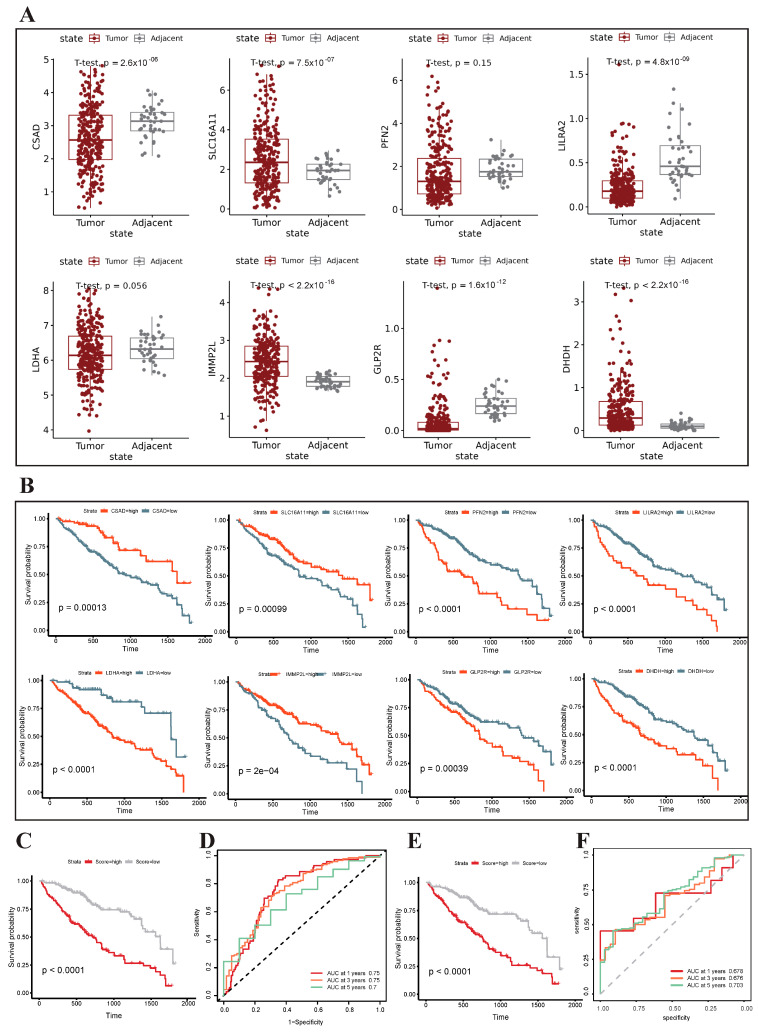
Construction and validation of the metabolism-related prognostic risk model. (**A**) Boxplot of the expression of eight characteristic genes in liver cancer and normal tissues. (**B**) Survival analysis for eight characteristic genes. (**C**,**E**) Survival curves for test set and validation set. (**D**,**F**) The AUC for the prediction of 1-, 3-, and 5-year survival rates in the test set and validation set.

By integrating tumor cell marker genes, marker genes of metabolic pathways enriched in malignant cells, and differentially expressed genes, prognostic risk model candidate genes were established (Table S1). Subsequently, the TCGA-LIHC training cohort underwent single-factor Cox regression analysis, and the LASSO algorithm was used to screen out eight characteristic genes (*CSAD*, *SLC16A11*, *PFN2*, *LILRA2*, *LDHA*, *IMMP2L*, *GLP2R*, and *DHDH*) with the lowest cross-validation error. Among these, six genes were significantly differentially expressed between liver cancer tissue and adjacent normal tissue (*CSAD*, *SLC16A11*, *LILRA2*, *IMMP2L*, *GLP2R*, and *DHDH*) (Figure 7A). Survival analysis revealed a significant correlation between prognosis and the expression of these eight genes (Figure 7B). Specifically, elevated expression levels of *SLC16A11*, *PFN2*, *LILRA2*, *IMMP2L*, and *GLP2R* were linked to an unfavorable prognosis, while higher expression levels of *CSAD*, *LDHA*, and *DHDH* are associated with a more favorable prognosis. We used these eight genes to construct a prognostic risk model (risk score = 4.165386 × 10^−2^ × *LDHA* + 1.029364 × 10^−2^ × *DHDH* − 7.596543 × 10^−3^ × *CSAD* − 7.964147 × 10^−3^ × *SLC16A11* + 1.141681 × 10^−2^ × *PFN2* − 1.178891 × 10^−2^ × *IMMP2L* + 6.160969 × 10^−5^ × *LILRA2* + 6.412018 × 10^−2^ × *GLP2R*). All the samples were divided into high-risk and low-risk groups based on a median risk score of 0.22. The survival curve showed that compared with samples in the low-risk group, the high-risk group had significantly lower overall survival (OS) (*p* < 0.0001) (Figure 7C,E). In order to further evaluate the effectiveness of the risk model, the ROC curve of OS was calculated. The AUC values of 1 year, 3 years and 5 years in the internal test set were all greater than 0.7, and the AUC values of 1 year, 3 years and 5 years in the external test set were also approximately 0.7, indicating high accuracy of the risk model (Figure 7D,F).

### 3.7. GSEA Analysis and Clinical Correlation between the High- and Low-Risk Groups

**Figure 8:**
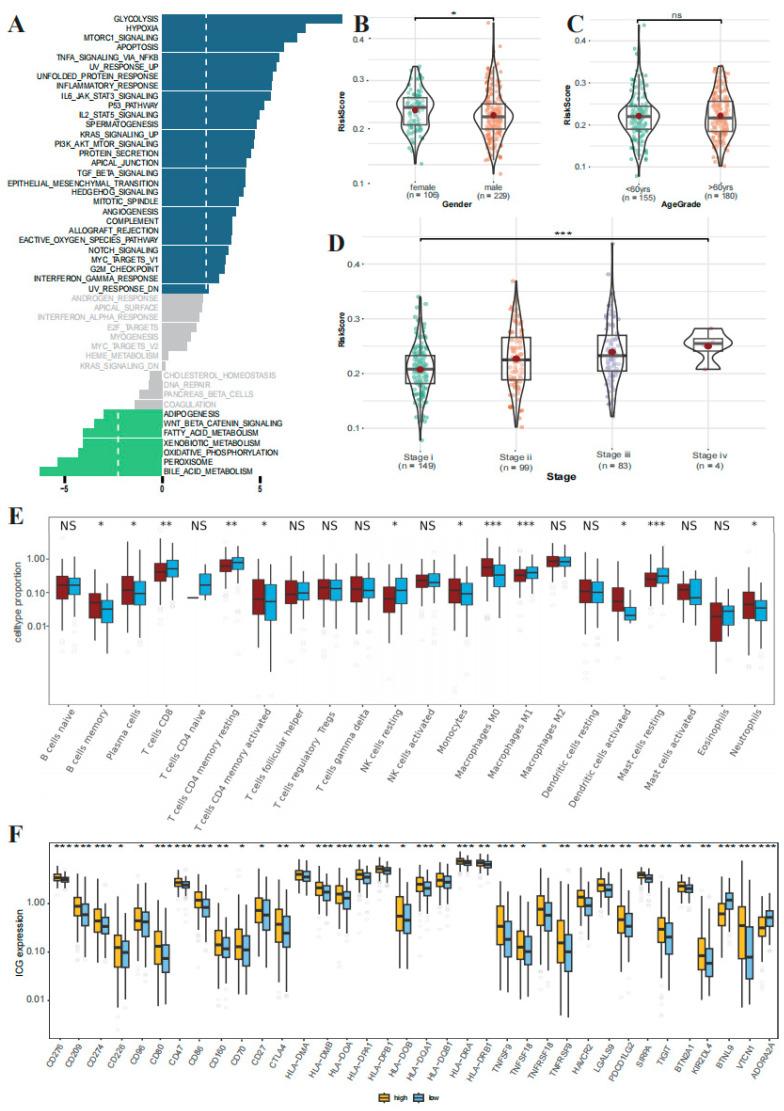
GSEA, immune cell infiltration, and immune checkpoint expression between the high- and low-risk groups. (**A**) GSEA of the high- and low-risk groups divided by prognostic risk model. (**B**–**D**) Association of the risk score with sex, age, and tumor stage. (**E**) Analysis of immune cell infiltration in the high- and low-risk groups (NS: *p* > 0.05, *: *p* < 0.05, **: *p* < 0.01, ***: *p* < 0.001). (**F**) Analysis of immune checkpoints between the high- and low-risk groups (*: *p* < 0.05, **: *p* < 0.01, ***: *p* < 0.001).

To investigate the differences between high-risk and low-risk groups in liver cancer progression, we performed GSEA analysis to identify pathways significantly enriched in the two groups. The results show that the high-risk group was enriched mainly in signaling pathways related to hypoxia, glycolysis, and inflammation, while the low-risk group was primarily enriched in metabolic pathways related to fatty acid metabolism and oxidative phosphorylation (Figure 8A). To explore the association between the risk score and clinical characteristics, we compared differences in prognostic risk scores according to clinical characteristics. Our findings revealed that males exhibited higher risk scores compared to females (Figure 8B), and we observed no statistically significant association between age and risk score categorization (Figure 8C). Notably, the risk score of the prognostic risk model increased significantly with increasing tumor stage (Figure 8D).

### 3.8. The Correlations Immune Cell Infiltration and Immune Checkpoint Expression between the High- and Low-Risk Groups

To further explore the correlation between risk score and immune status, we analyzed the differences in tumor microenvironment between high-risk and low-risk groups. A comprehensive screening of 22 tumor-infiltrating immune cells was performed. The results showed that the infiltration scores of memory B cells, plasma cells, activated memory CD4 T cells, monocytes, M0 macrophages, activated dendritic cells, and neutrophils were greater in the high-risk group (*p* < 0.05). The infiltration scores of CD8 T cells, resting memory CD4 T cells, resting NK cell, M1 macrophages, and resting mast cell were greater in the low-risk group (*p* < 0.05) (Figure 8E). Furthermore, we conducted an examination of disparities in immune checkpoint expression between the high-risk and low-risk groups. The results showed that a total of 36 immune checkpoints were significantly differentially expressed in high- and low-risk groups. Except for *BTNL9* and *ADORA2A*, which had lower expression in the high-risk group, the other 34 immune checkpoint genes were highly expressed in the high-risk group (*p* < 0.05) (Figure 8F). In conclusion, the high-risk group manifests heightened activation of diverse immune cells, coupled with elevated expression levels of multiple immune checkpoints, which is indicative of a more intricate tumor microenvironment. This finding implies a potentially heightened responsiveness to immunotherapy in the high-risk group.

### 3.9. Drug Sensitivity Analysis of the High-Risk and Low-Risk Groups

**Figure 9:**
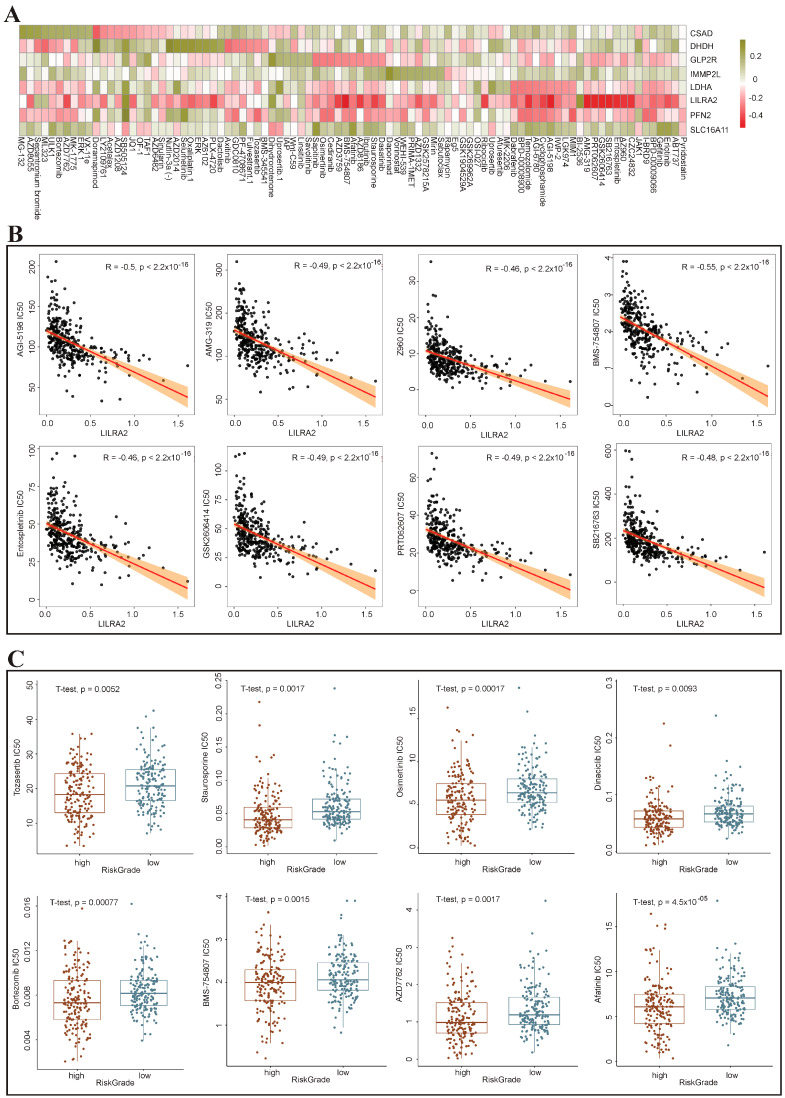
Drug sensitivity analysis of the high- and low-risk groups. (**A**) Chemotherapy drug enrichment analysis of eight prognostic genes. (**B**) The top eight most relevant gene drugs. (**C**) Boxplot of targeted drugs with lower IC50 values for high-risk groups.

To prognosticate the clinical responsiveness to targeted therapy in high-risk liver cancer patients, we analyzed the correlation between eight prognostic risk genes and chemotherapy drugs. The heatmap revealed a significant correlation between the *LILRA2* and the majority of chemotherapy drugs (Figure 9A). Simultaneously, we ranked the genes based on the correlation between gene-drug pairs. Notably, the top eight genes exclusively included *LILRA2*, showing a marked negative correlation (Figure 9B). The expression of *LILRA2* gene is lower in liver cancer patients, suggesting that the low expression of this gene in such patients may be a pivotal factor contributing to their insensitivity to chemotherapy drugs and poor response to targeted therapy. Subsequently, we conducted a more in-depth analysis of the correlation between the prognostic model risk score and targeted drugs. It was observed that afatinib, AZD7762, BMS-754807, bortezomib, dinaciclib, osimertinib, staurosporine, and tozasertib had lower IC50 values within the high-risk group (Figure 9C). This finding underscores that the prognostic risk model grounded in the metabolic genes of liver cancer malignant cells demonstrates efficacy in predicting both the effectiveness and sensitivity of chemotherapy. Furthermore, this approach enhances the clinical viability of precision treatment for liver cancer guided by drug screening.

## 4. Discussion

Liver cancer stands out as an exceptionally diverse form of malignant disease compared to the tumors identified thus far [42]. Despite many recent efforts in the treatment of liver cancer, its heterogeneous and aggressive characteristics remain limited in prognostic evaluation [5]. Consequently, the identification of novel biomarkers that facilitate the advancement of patient-specific therapies and enhance patients’ prognosis is crucial and imperative. In contrast to bulk RNA-seq, which conventionally centers on the mean expression levels of genes within cells, scRNA-seq has emerged as a potent approach for delineating the transcriptomic profiles of cell subpopulations. Hence, in this study, we conducted a comprehensive analysis of bulk RNA-seq and scRNA-seq of liver cancer, and constructed a liver cancer prognostic risk model from the perspective of tumor cell metabolism, the results demonstrated favorable prognostic and immunotherapy responses in liver cancer patients.

First, we identified 11 core cells, including malignant cells, fibroblasts, macrophages, endothelial cells, B cells, plasma cells, gammadelta T cells, and 4 types of T cell subtypes, in a scRNA-seq profile containing 105,829 cells. Through the extraction and subsequent re-clustering of neoplastic cells, we discerned nine subclusters, revealing a pronounced degree of inter-tumoral heterogeneity among the neoplastic cells. Our investigation revealed notably heightened metabolic activity within malignant cells, with a particularly conspicuous up-regulation observed in the metabolic pathways of subclusters 2 and 8. Furthermore, pseudotime trajectory analysis revealed the positioning of subclusters 2 and 8 at the terminal phase of differentiation, revealing that malignant cells in hepatocellular carcinoma undergo metabolic reprogramming events during the concluding stages of differentiation to sustain neoplastic proliferation and metastatic potential. Moreover, through cell–cell interaction analysis, we discerned a heightened level of interaction intensity between malignant cells and other cellular entities. Particularly notable is the pronounced abundance of interactions within the MIF/CD74 axis involving malignant cells and their counterparts. Notably, malignant cells emerge as the principal conveyors of signals within the MIF signaling pathway.

Next, taking tumor cell marker genes, tumor cell enriched metabolic pathway marker genes and differentially expressed genes as candidate genes, a prognostic risk model based on eight genes was established through univariate Cox regression analysis and the LASSO algorithm. The results of the ROC curve indicate a favorable predictive efficacy of the model for prognosis. After validation with an external dataset, the AUC was approximately 0.7. Upon comparing patient risk scores across distinct clinical cohorts, it was discerned that males exhibited elevated risk scores in comparison to females. Furthermore, risk scores derived from the prognostic risk model demonstrated a significant increase in tandem with increased tumor grade and stage. In summary, the prognostic risk model has a remarkably favorable predictive ability.

Finally, we analyzed the immune status and drug sensitivity of the high-risk and low-risk groups divided by prognostic risk model. The results showed that the high-risk group had a more active immune cell status and higher expression of immune checkpoints, reflecting its more complex tumor microenvironment. Moreover, through correlation analysis between prognostic risk genes and chemotherapeutic drugs, we found that low expression of *LILRA2* in liver cancer patients may be an important reason for their insensitivity to chemotherapeutic drugs and poor therapeutic efficacy. In addition, we identified potential liver cancer-targeting drugs for seeding (afatinib, bortezomib, dinaciclib and osimertinib, etc.). Although these drugs have not been widely adopted clinically due to their potency and promiscuity, as technology is updated, they will become highly promising anti-tumor drugs in the future.

The advantage of this work lies in its construction of a novel prognostic risk model that comprehensively considers diverse factors, including tumor heterogeneity, cellular interactions, tumor cell metabolism, and differentially expressed genes. This model demonstrated proficiency in precisely discriminating between survival outcomes and immunotherapy responses in patients with liver cancer. The outcomes of this investigation provide conclusive evidence supporting the stratification and meticulous treatment of patients afflicted with liver cancer. Nevertheless, this study is not without inevitable limitations: (1) the sample size of the scRNA-seq data was relatively small; (2) the regulatory mechanism of characteristic genes in liver cancer is still unclear, and the re-clustering of malignant cells gives rise to subclusters, necessitating further exploration. This signifies a path that warrants continued investigation in subsequent phases of this study.

## 5. Conclusions

Starting from the construction of a liver cancer single-cell profile and combining bulk RNA-seq data, we focused our research on the tumor cell metabolism in liver cancer. Employing machine learning methodologies, we formulated a novel prognostic risk model tailored to liver cancer patients. Additionally, through GSEA, clinical correlation assessments, immune infiltration and immune checkpoint correlations, and drug sensitivity analyses for high- and low-risk cohorts in liver cancer, our findings elucidate notable disparities in the interplay between immune status and drug responsiveness, as projected by the model. Moreover, our investigations unveiled the potential significance of the *LILRA2* gene as a pivotal target for liver cancer immunotherapy. In summary, this inquiry serves as a reliable prognosticator of liver cancer efficacy, thereby paving the way for innovative approaches in the targeted treatment landscape of liver cancer.

## Data Availability

The original contributions presented in the study are included in the article/Supplementary Material, further inquiries can be directed to the corresponding authors.

## Supplementary Materials

The following supporting information can be downloaded at: https://www.mdpi.com/article/10.3390/genes15060755/s1, Table S1: Prognostic model candidate gene set.
